# Detection of gastric cancer with implantation metastasis to the inguinal region via ^18^F-FAPI PET/CT: a case report

**DOI:** 10.3389/fonc.2025.1549825

**Published:** 2025-07-08

**Authors:** Haibo Zhang, Shanhu Hao, Tong Wu, Zhongbao Wu, Xuantong Liu, Ying Xu, Peng Zhang, Ying Yan

**Affiliations:** ^1^ Department of Radiation Oncology, General Hospital of Northern Theater Command, Shenyang, Liaoning, China; ^2^ Department of Nuclear Medicine, General Hospital of Northern Theater Command, Shenyang, Liaoning, China; ^3^ Department of Pathology, General Hospital of Northern Theater Command, Shenyang, Liaoning, China

**Keywords:** gastric cancer, metastasis, PET/CT, FAPI, radiotherapy

## Abstract

Implantation metastasis of gastric cancer to the inguinal area is rarely reported, and a clear diagnosis of such metastasis is hard to make, especially when these cancers are invisible on fluorodeoxyglucose (FDG) positron emission tomography/computed tomography (PET/CT) imaging. However, certain types of cancers, such as signet-ring cell carcinoma, are particularly sensitive to fibroblast activation protein inhibitor (FAPI) PET/CT. In this case, a patient with a history of gastric cancer and a gastrectomy 2 years previously suddenly developed serious edema in his right lower limb. With the help of FAPI PET/CT and other laboratory findings, abdominal implantation of his primary gastric cancer to the inguinal region was confirmed. After prompt treatment, the patient had a favorable prognosis.

## Introduction

Since distant metastasis of gastric cancer is regarded as one of the most critical risk factors associated with patients’ prognosis, several common patterns of metastasis, including lymphatic metastasis, hematogenous metastasis, and peritoneal implantation, have been intensively studied. Implantation metastasis to the groin, which is separated from visceral organs by the peritoneum, is rarely observed. In terms of diagnosis of these malignancies, fluorodeoxyglucose (FDG) positron emission tomography/computed tomography (PET/CT) is usually the first choice due to high glucose intake of these malignancies, while some types of gastric cancer, such as signet-ring cell carcinoma and mucinous adenocarcinoma, are more likely to be visualized by fibroblast activation protein inhibitor (FAPI) PET/CT rather than FDG PET/CT (1, 2). According to some reports, FAPI PET/CT has been used for several tumors (3–5).

## Case report

**Figure 1:**
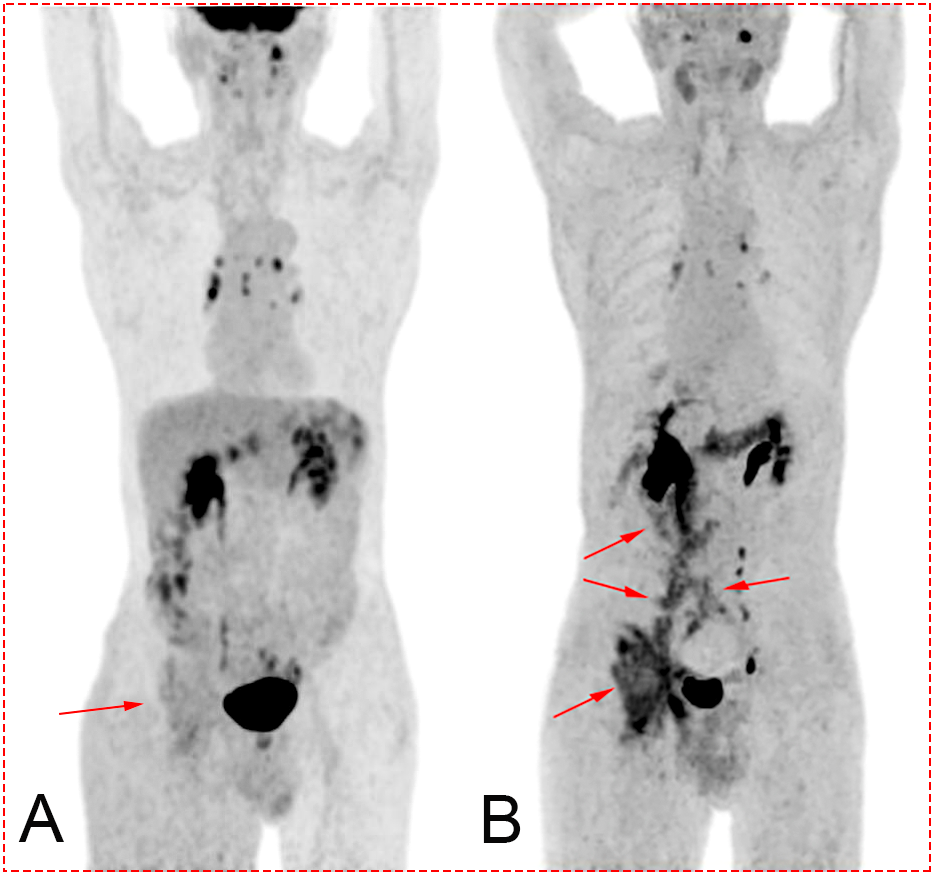
**(A)** Maximum intensity projection (MIP) after ^18^F-FDG PET/CT. Image of the tumor is not clearly displayed (red arrow). **(B)** MIP after ^18^F-FAPI PET/CT. The darker sites labeled by the red arrow show the path of metastasis of the tumor from the tumor bed (duodenal stump) to the right groin. Concentration of the tracer mainly located in the biliary tract, pelvis, ureter, bladder, pancreas, and colon.

**Figure 2:**
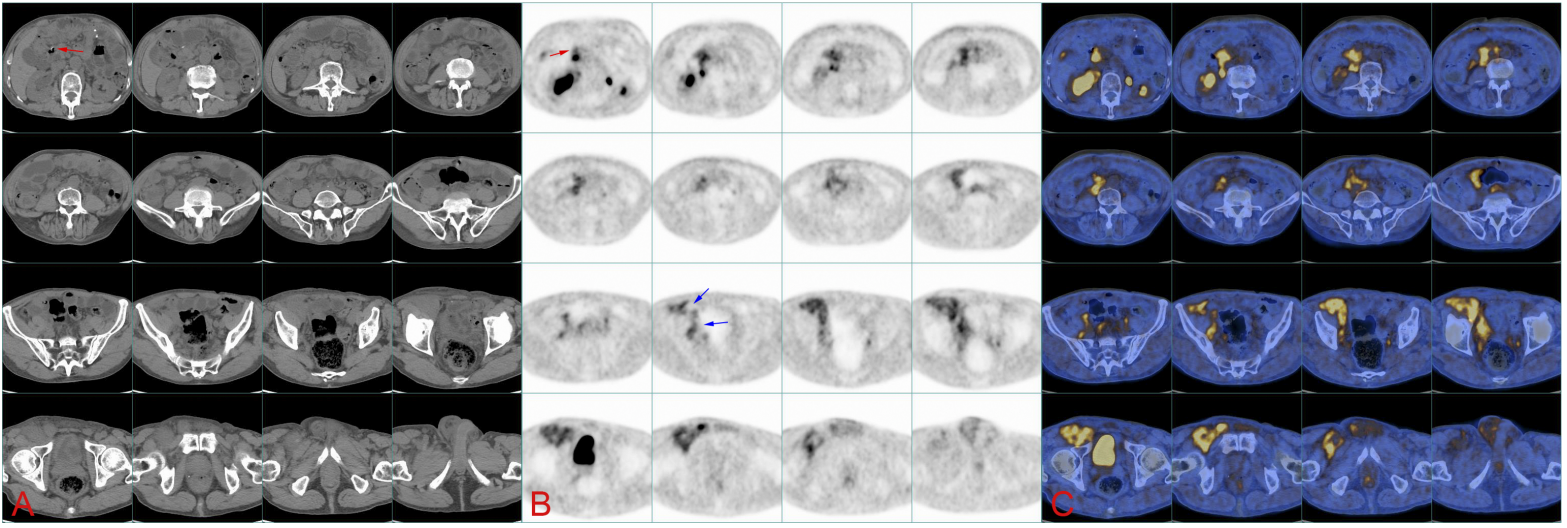
**(A)** Continuous CT images of the abdomen show the location of soft tissue on the right pelvic wall and groin, while the image of tumor in the abdomen is not clear. Red arrows point at the duodenal stump. **(B)** ^18^F-FAPI PET/CT image. The red arrow indicates the duodenal stump (primary tumor bed), and blue arrows indicate the tumor flowing through the iliac blood vessels into the groin. **(C)** ^18^F-FAPI PET/CT fusion image.

**Figure 3:**
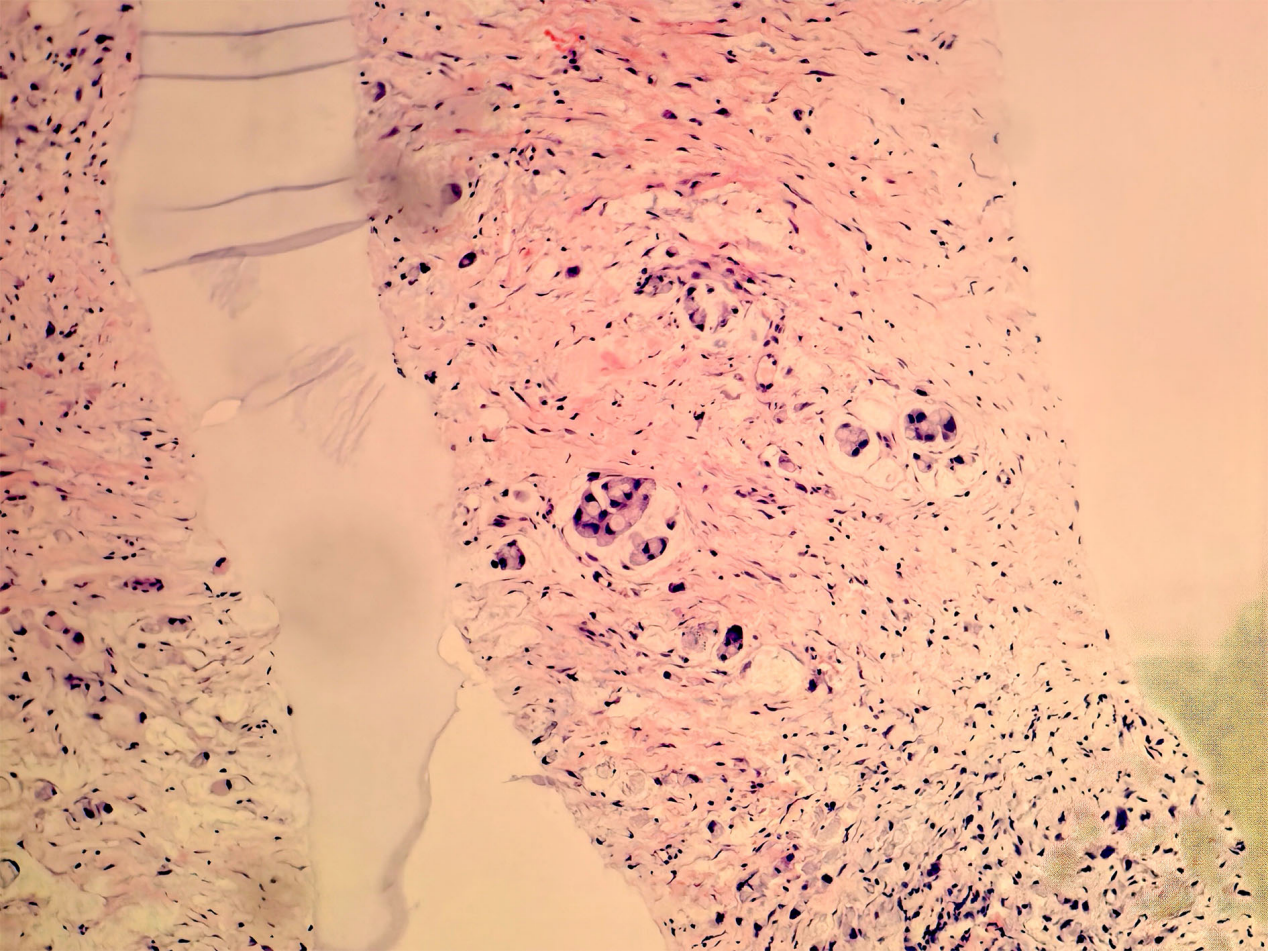
Biopsy of soft tissue at the right groin. Heterotypic cells were observed via immunohistochemistry, suggesting the diagnosis of a gastrointestinal metastatic adenocarcinoma.

**Figure 4:**
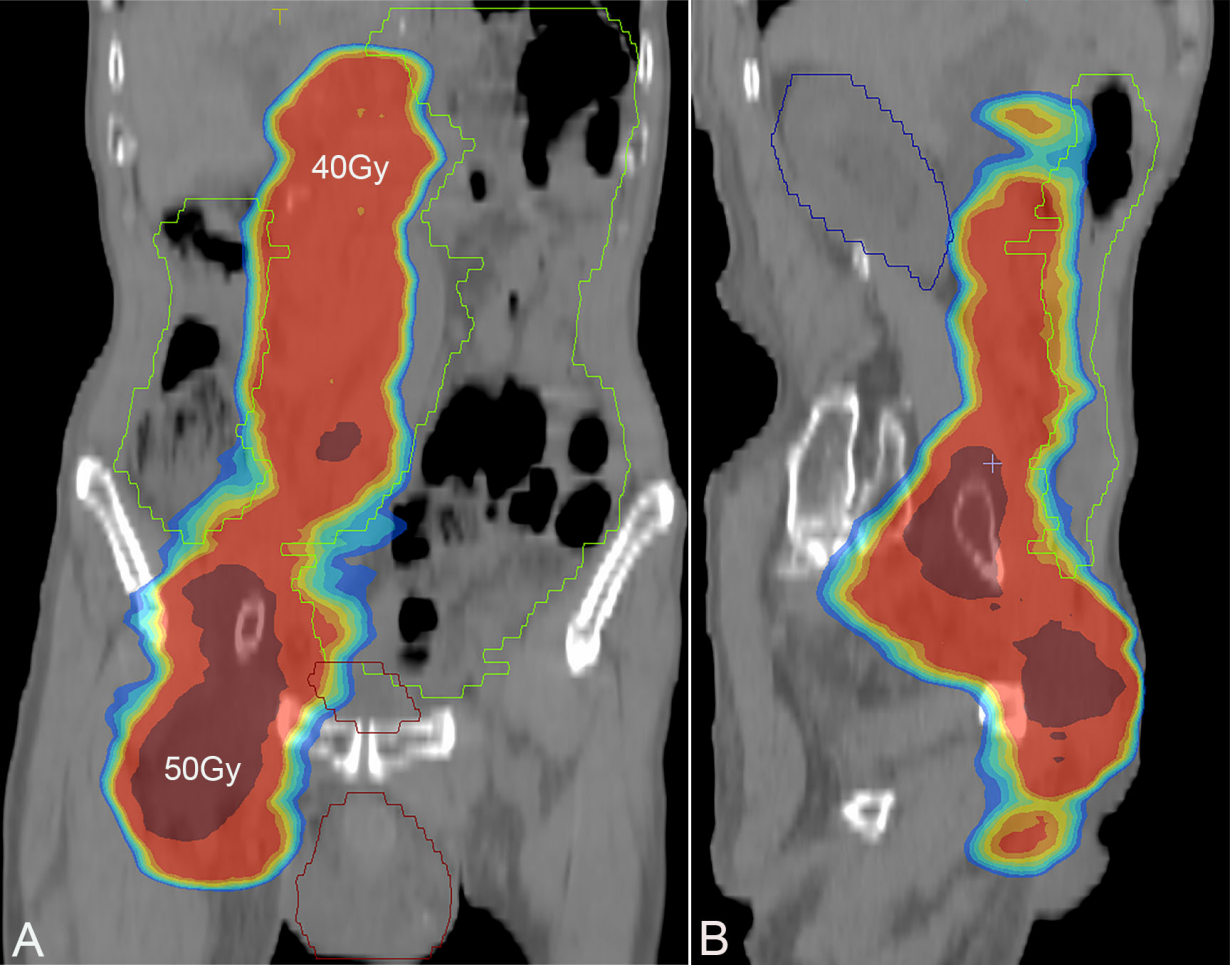
Screenshot of radiotherapy plan, with a dose of 40 Gy in the bright red area and a dose of 50 Gy in the deep red area. **(A)** Coronal plane; **(B)** sagittal plane.

**Figure 5:**
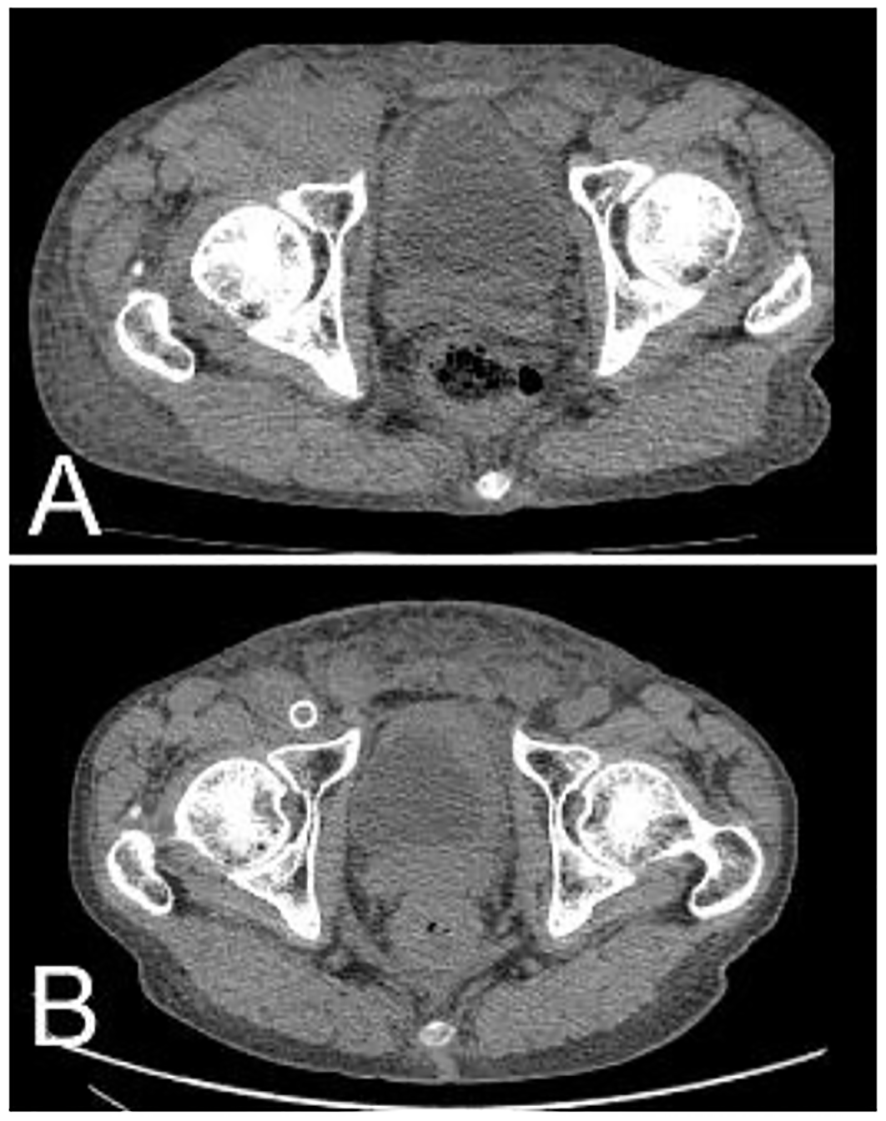
After radiotherapy and chemotherapy, the right inguinal tumor shrank significantly. **(A)** Pretreatment; **(B)** 1 year after treatment.

A 78-year-old man was admitted to our hospital with a complaint of right lower extremity swelling for 3 months. Two years ago, he underwent a gastroscopy that revealed a gastric antral tumor, and subsequent pathological findings indicated a poorly differentiated adenocarcinoma with partial signet-ring cell. Distal subtotal Billroth II gastrectomy and D2 lymphadenectomy were then performed. Postoperative pathology indicated a moderately to poorly differentiated ulcerated adenocarcinoma with infiltration into the serosa, invasion of nerves, and lymph node metastasis (3/39). Two cycles of CAPOX chemotherapy were given after the operation but discontinued due to severe side effects. The patient did not have any other past medical history. Physical examination showed severe pitting edema confined to his right lower extremity. No abnormal abdominal mass was found except for a visible surgical incision. Laboratory tests showed slightly decreased leukocyte (3.2 × 10^9^/L, normal: 3.5–9.5), hemoglobin (96 g/L, normal: 130–175), and serum sodium (133.4 mmol/L, normal: 137–147), as well as elevated D-dimer (1.23 mg/L), Carcinoembryonic antigen (CEA) (16.84 ng/mL, normal: 0–4.5), CA199 (410.79 U/mL, normal: 0–37), CA724 (222.16 U/mL, normal: 0–6), and CA50 (>180 U/mL, normal: 0–25). Other indexes were within normal ranges. B-ultrasound examination revealed a marked swelling of perivascular soft tissue in the right inguinal region. Compression of the right common femoral vein and slow blood flow were also seen. A total abdominal CT examination indicated that there were soft tissues beside the external iliac blood vessels on the right pelvic wall, suggesting the possibility of enlarged lymph nodes. Right hydroureter at the proximal part and right hydronephrosis were observed, and the right pelvic ureter was also suspected to be involved. Slight pelvic cavity effusion and multiple sites of exudation in the subcutaneous tissue of the abdomen were also observed. In order to identify the cause of his right lower extremity swelling, the patient underwent a ^18^F-FDG PET/CT examination (**Figure 1A**), and no clear signs of increased metabolism in the right pelvic wall and groin were seen. Considering that the patient had been diagnosed with gastric cancer that consisted partially of signet-ring cells, ^18^F-FAPI PET/CT examination was performed instead. As a result, increased metabolism of the swelling soft tissues in the right pelvic wall and groin was observed. In addition, these areas with high metabolism originated from the duodenal stump and extended down through the abdominal compartment to the right pelvic wall and groin (**Figures 1B**, **2**). Such evidence suggested that the patient’s primary tumor may have experienced implantation metastasis to the right groin. Soft tissue biopsy guided by B-ultrasound was then performed in the right groin and, histologically, evidence of a gastrointestinal metastatic adenocarcinoma was confirmed (**Figure 3**). For treatment, anterograde venography of both lower limbs was first carried out, and the imaging showed some small areas of filling defect in the right calf intermuscular vein, while the popliteal vein and superficial femoral vein were not involved. Meanwhile, serious stenosis of the right common femoral vein and right external iliac vein were observed, along with a slower blood flow, but without any thrombus, considered to be caused by some external pressure on the vascular wall. Imaging of the left limb showed no abnormality. Subsequently, balloon dilation (Mindstorm ev3 of America, Plymouth, Minnesota, USA) and stent implantation on the right external iliac vein and right common femoral vein were performed with the placement of two self-expandable stents (W. L. Gore & Associates, Flagstaff, Arizona, USA and Mindstorm ev3 of America, Plymouth, Minnesota, USA). After the above interventional therapy, edema of the patient’s right lower limb was gradually relieved. Palliative radiotherapy was then administered to the lesion area at a dose of 40 Gy in 20 fractions over 4 weeks and simultaneously to the right pelvic wall and groin with a dose of 50 Gy in 20 fractions over 4 weeks (**Figure 4**). In terms of chemotherapy, the CapeOX regimen was meanwhile carried out for one cycle, followed by maintenance therapy with oral capecitabine monotherapy. After 1 year of follow-up, the tumor was effectively relieved, and the efficacy evaluation indicated partial remission (PR) (response evaluation criteria in solid tumors 1.1) (**Figure 5**). Normal life and physical activities resumed, with a Zubrod performance status (ZPS) score of 1.

## Discussion

Metastasis of gastric cancer in the inguinal region has been rarely reported due to the absence of an obvious anatomical pathway between the abdominal cavity and the inguinal area. Although cases of gastric cancer with metastasis to the skin at the inguinal region and subcutaneous tissue of the scrotum have been described before (6, 7), none of the reports has mentioned a pattern of metastasis that directly implants into the groin. In this case, a patient with a history of gastric cancer and gastrectomy initially presented with lower limb edema, and on subsequent CT and ultrasound, swelling of soft tissue instead of a well-defined mass was observed around the inguinal vessels. Furthermore, FDG PET/CT failed to visualize conspicuous hypermetabolism in the groin, which made the diagnosis of metastatic cancer particularly difficult. Nevertheless, by employing FAPI PET/CT, it was confirmed that the primary tumor that originated from the tumor bed (duodenal stump) extended through the mesenteric interval within the abdominal cavity to spaces around the right iliac vessels, flowing along the wall of these vessels until the inguinal vascular region, thus forming a large soft tissue mass, which compressed the right external iliac vein and right common femoral vein and thereby caused lower extremity edema.

FDG remains the most widely used PET/CT tracer in clinical practice because of the high glucose metabolism in most tumors, while certain types of cancers, such as in this case report, do not exhibit elevated FDG uptake, which may hinder accurate diagnosis and staging. However, according to some reports and the above case, signet-ring cell gastric carcinoma, which contains cancer-associated fibroblasts that express fibroblast activation protein (FAP), can be well-detected by FAPI PET/CT, demonstrating enhanced uptake compared with FDG-PET (1, 2, 5). The atypical metastatic site in this case has enabled the employment of FAPI PET/CT over conventional diagnostic approaches. Such imaging techniques have offered a “chain of evidence” to clarify the metastatic route of a primary tumor and may establish a robust foundation for future research into more possible metastatic patterns of gastric cancer.

## Data Availability

The original contributions presented in the study are included in the article/supplementary material. Further inquiries can be directed to the corresponding author.
